# Conditional Dependence between Response Time and Accuracy: An Overview of its Possible Sources and Directions for Distinguishing between Them

**DOI:** 10.3389/fpsyg.2017.00202

**Published:** 2017-02-16

**Authors:** Maria Bolsinova, Jesper Tijmstra, Dylan Molenaar, Paul De Boeck

**Affiliations:** ^1^Department of Psychology, University of AmsterdamAmsterdam, Netherlands; ^2^Department of Methodology and Statistics, Tilburg UniversityTilburg, Netherlands; ^3^Department of Psychology, Ohio State UniversityColumbus, OH, USA; ^4^Department of Psychology, KU LeuvenLeuven, Belgium

**Keywords:** conditional dependence, response processes, speed-accuracy trade-off, measurement, modeling response times

## Abstract

With the widespread use of computerized tests in educational measurement and cognitive psychology, registration of response times has become feasible in many applications. Considering these response times helps provide a more complete picture of the performance and characteristics of persons beyond what is available based on response accuracy alone. Statistical models such as the hierarchical model (van der Linden, 2007) have been proposed that jointly model response time and accuracy. However, these models make restrictive assumptions about the response processes (RPs) that may not be realistic in practice, such as the assumption that the association between response time and accuracy is fully explained by taking speed and ability into account (conditional independence). Assuming conditional independence forces one to ignore that many relevant individual differences may play a role in the RPs beyond overall speed and ability. In this paper, we critically consider the assumption of conditional independence and the important ways in which it may be violated in practice from a substantive perspective. We consider both conditional dependences that may arise when all persons attempt to solve the items in similar ways (homogeneous RPs) and those that may be due to persons differing in fundamental ways in how they deal with the items (heterogeneous processes). The paper provides an overview of what we can learn from observed conditional dependences. We argue that explaining and modeling these differences in the RPs is crucial to increase both the validity of measurement and our understanding of the relevant RPs.

## 1. Introduction

Using the statistical tools of for example item response theory (IRT; see e.g. Hambleton and Swaminathan, 1985; van der Linden and Hambleton, 1997), responses to items from cognitive and educational tests are used to make inferences about the underlying abilities. While standard IRT models attempt to capture quantitative differences in measured ability, these models focus only on the correctness of the responses (i.e., response accuracy; RA). When using these models, between- and within-person differences in response processes (e.g., used solution strategies, concentration, or operating speed) are taken to be noise and are usually ignored in practice, despite possibly being relevant for the assessment of persons. An important indicator of those possible differences in response processes (RPs) is response time (RT), which due to the increasing popularity of computerized testing has become available in many applications of educational and cognitive testing. Considering this additional information provides a more complete picture of the RPs.

Within psychometrics effort has been devoted to developing suitable joint models for RT and RA to incorporate this additional source of information into the traditional measurement procedures (e.g., Thissen, 1983; van der Linden, 2007; Molenaar et al., 2015a,b). The hierarchical modeling framework (van der Linden, 2007, 2009) presents a theoretically appealing approach for making use of RT in ability measurement, and has arguably become the dominant approach to modeling RA and RT in educational measurement. It posits two measurement models: one for RA, capturing a person's effective ability (θ), and one for RT, capturing a person's effective speed (τ). The two models are linked at the population level, where both the speed and ability of persons, and the difficulty and time intensity of items can be correlated.

**Figure 1:**
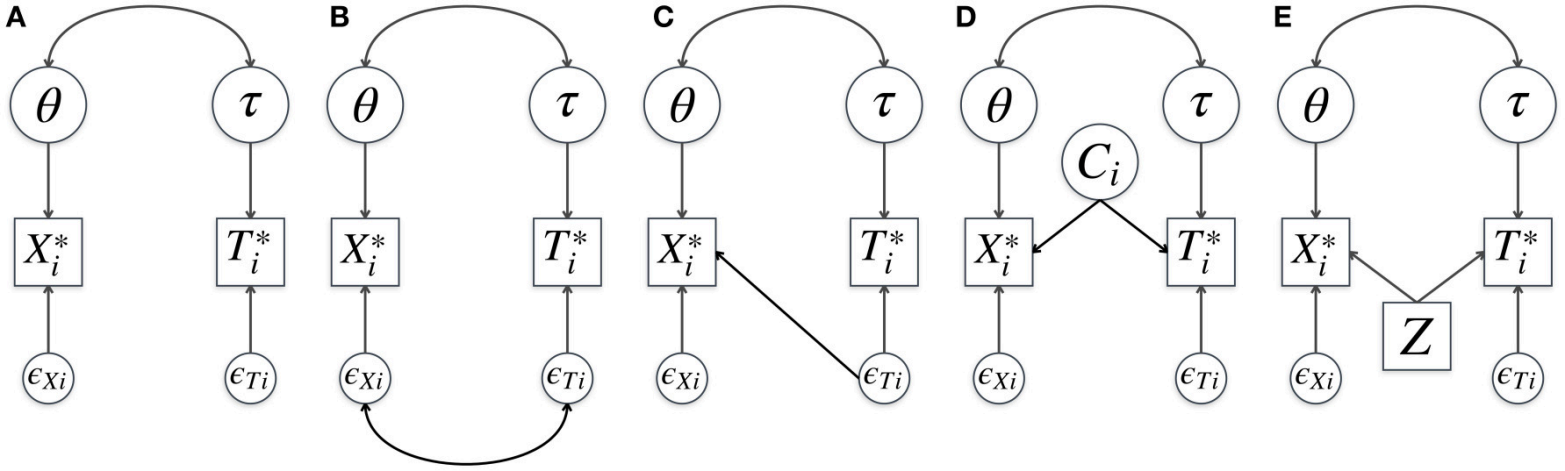
**Joint models for response time and accuracy:** standard hierarchical model **(A)**, existing descriptive models for conditional dependence **(B–D)**, and an example of an explanatory model for conditional dependence **(E)**. θ and τ are speed and ability, $X_{i}^{*}$ and $T_{i}^{*}$ are continuous underlying response accuracy and log-transformed response time of item *i*, ϵ_*Xi*_ and ϵ_*Ti*_ are the residuals, *C*_*i*_ is a latent class of a response to item *i*, and *Z* is a covariate (e.g., school type). In **(A)** conditional independence given θ and τ is assumed. In **(B)** the correlation between the residuals of time and accuracy is added to the model (Ranger and Ortner, 2012; Meng et al., 2015). In **(C)** an effect of residual response time on response accuracy is modeled (Bolsinova et al., 2016a,b). In **(D)** different latent classes of responses are considered which differ both in response time and accuracy (Molenaar et al., 2016b). In **(E)** possible conditional dependence between time and accuracy given θ and τ is explained by an observed covariate *Z*.

One of the central questions when it comes to modeling RT and RA is about explaining the relationship between these two outcome measures. The hierarchical model (HM) assumes the association between RT and RA to be fully explained by the correlation between the person characteristics and between the item characteristics: it assumes *conditional independence* (CI) of RA and RT given those person and item characteristics^1^. This is graphically illustrated in Figure 1A. However, in real-life applications there may be important residual associations between RT and RA, indicating misfit of the HM. These conditional dependences (CDs) might be relevant from a substantive point of view since they shed light on the interesting phenomena in RPs and possible between- and within-person differences.

Recently, attention has been devoted to testing the CI assumption (van der Linden and Glas, 2010; Bolsinova and Maris, 2016; Bolsinova and Tijmstra, 2016) and joint models for RT and RA have been extended to account for CD (Ranger and Ortner, 2012; Meng et al., 2015; Bolsinova et al., 2016a,b; Molenaar et al., 2016b), see Figures 1B–D. While different in their approach to CD, these models share the feature that they deal with the dependence in a descriptive way. That is, the dependence is captured by the model, but not explained by it (e.g., it is not explained why some items have positive CD and some have negative CD, see Figures 1B,C, or what different classes of responses represent, see Figure 1D). In this paper, instead of taking a statistical point of view we are looking at CD from a more substantive and explanatory perspective and discuss the different kinds of RP phenomena that may lead to CD. Taking these phenomena into consideration (and ideally including them in the statistical model if they are present) is important for two reasons: (1) to improve the quality of the measurement of the attributes of interest and (2) to try to learn about the cognitive processes leading to the responses.

Including RT in measurement models forces one to focus on aspects of RPs that without RT are convenient or at least easy to ignore, that is, the possible confounding of measurement due to differences in RPs. As will be elaborated in the subsequent sections, these relevant differences in the RPs may be qualitative in nature (i.e., heterogeneous RPs), but can also be present when the RPs do not differ fundamentally (i.e., homogeneous RPs). In this paper, we take a careful look at the substantive assumptions about the RPs that are made by common joint models for RA and RT, and explore the ways in which these assumptions may be violated in practice. These assumptions are the following: (1) there is no systematic within-person variation of speed and ability across items; (2) item characteristics are constant across persons; (3) responses come from the same process.

The paper is organized as follows. In Section 2 we discuss how CD can arise when assumptions 1 and 2 are violated in cases of homogeneous RPs. In Section 3 we elaborate on heterogeneous RPs (i.e., persons do different things when confronted with an item), which entail a violation of assumption 3 and potentially cause CD. The paper concludes with a discussion on what steps one could take to distinguish the different possible phenomena presented in this paper in practice.

## 2. Homogeneous response processes

Let us without loss of generality consider a simple specification of the HM assuming CI with the one-parameter normal ogive model for RA and the log-normal model for RT (van der Linden, 2006):

(1) $$\begin{array}{l} \left[\begin{array}{c} X_{pi}^{*}\\ T_{pi}^{*} \end{array}\right]\sim N_{2}\left(\left[\begin{array}{c} \theta_{p}-\beta_{i}\\ \xi_{i}-\tau_{p} \end{array}\right],\left[\begin{array}{cc} 1 & 0\\ 0 & \sigma_{i}^{2} \end{array}\right]\right), \end{array}$$

where $X_{pi}^{*}$ and $T_{pi}^{*}$ are the underlying continuous RA and the log-transformed RT of person *p* on item *i*, respectively; and β_*i*_, ξ_*i*_, and $\sigma_{i}^{2}$ are the difficulty, the time intensity and the residual variance of item *i*, respectively. $X_{i}^{*}$ and $T_{i}^{*}$ can be both represented as the sum of their expected value and the normally distributed residuals. A nonzero correlation between these two residuals constitutes CD, while a zero correlation implies that CI holds.

The residual RT can be partly due to a fluctuation of τ (i.e., a person working relatively faster or slower on an item than on the test as a whole) or a fluctuation of ξ_*i*_ (i.e., an item being relatively more or less time intensive for a person than for the average person). Analogously, part of the residual RA can be a fluctuation of θ (i.e., persons performing above or below their average performance on the test) or a fluctuation of β_*i*_ (i.e., an item being relatively more or less difficult for this particular person compared to other persons). In the traditional HM these fluctuations are taken to be noise and the fluctuations on the RA and the RT sides are taken to be uncorrelated. However, in practice θ_*p*_ and τ_*p*_ might co-vary across items, and β_*i*_ and ξ_*i*_ might co-vary across persons.

It may be noted that statistically speaking the variation of β_*i*_ and ξ_*i*_ across persons cannot be disentangled from the variation of θ and τ of persons across items. They are the two sides of the same person-by-item interaction. That is, instead of saying that β_*i*_ and ξ_*i*_ are higher for person *p* than expected, we might say that θ_*p*_ and τ_*p*_ are lower than expected when responding to item *i*. However, these two phenomena are conceptually different as will be elaborated in the next subsections and it is important to consider them separately when trying to understand RPs.

### 2.1. Variation of speed and ability of a person across items

The within-person relationship between speed and accuracy has been of primary interest for cognitive psychologists (Townsend and Ashby, 1983; Luce, 1986). The phenomenon that RA generally decreases as a person increases their speed (speed-accuracy trade-off, SAT; for an overview see e.g., Heitz, 2014) has been studied extensively and is well-established in a variety of cognitive tasks, such as memory retrieval (Reed, 1973; Dosher, 1976), visual search (e.g., McElree and Carrasco, 1999; Carrasco and McElree, 2001), and perceptual decision making (e.g., Kleinsorge, 2001; Wenzlaff et al., 2011). SAT is studied using different experimental methods, such as varying deadlines or varying the time when participant are given a signal to respond (for an overview of methods, see e.g., Wickelgren, 1977).

While usually not intended by the test developers, persons may still change their balance of speed and accuracy while taking a test. This may be especially plausible in settings where a strict total time limit is imposed, but could occur due to other factors as well. Such unmodeled variation in the response caution (i.e., the choice of speed-accuracy balance) within persons would result in positive CD given θ and τ.

The speed-accuracy trade-off paradigm takes as its starting point that persons have a constant amount of cognitive resources (cognitive capacity) at their disposal, which can be used either to work relatively fast but make many mistakes, or to work more accurately but slowly. However, cognitive capacity may also change throughout the test, for example if the level of motivation or the level of concentration changes (e.g., due to fatigue; Mollenkopf, 1950). In this case RT may decrease while RA increases if both concentration and motivation increase, or vice versa if both concentration and motivation decrease (i.e., negative CD). This relates to the person-specific variance of the drift rate in the diffusion model (Ratcliff, 1978), which if present also leads to negative CD.

**Figure 2:**
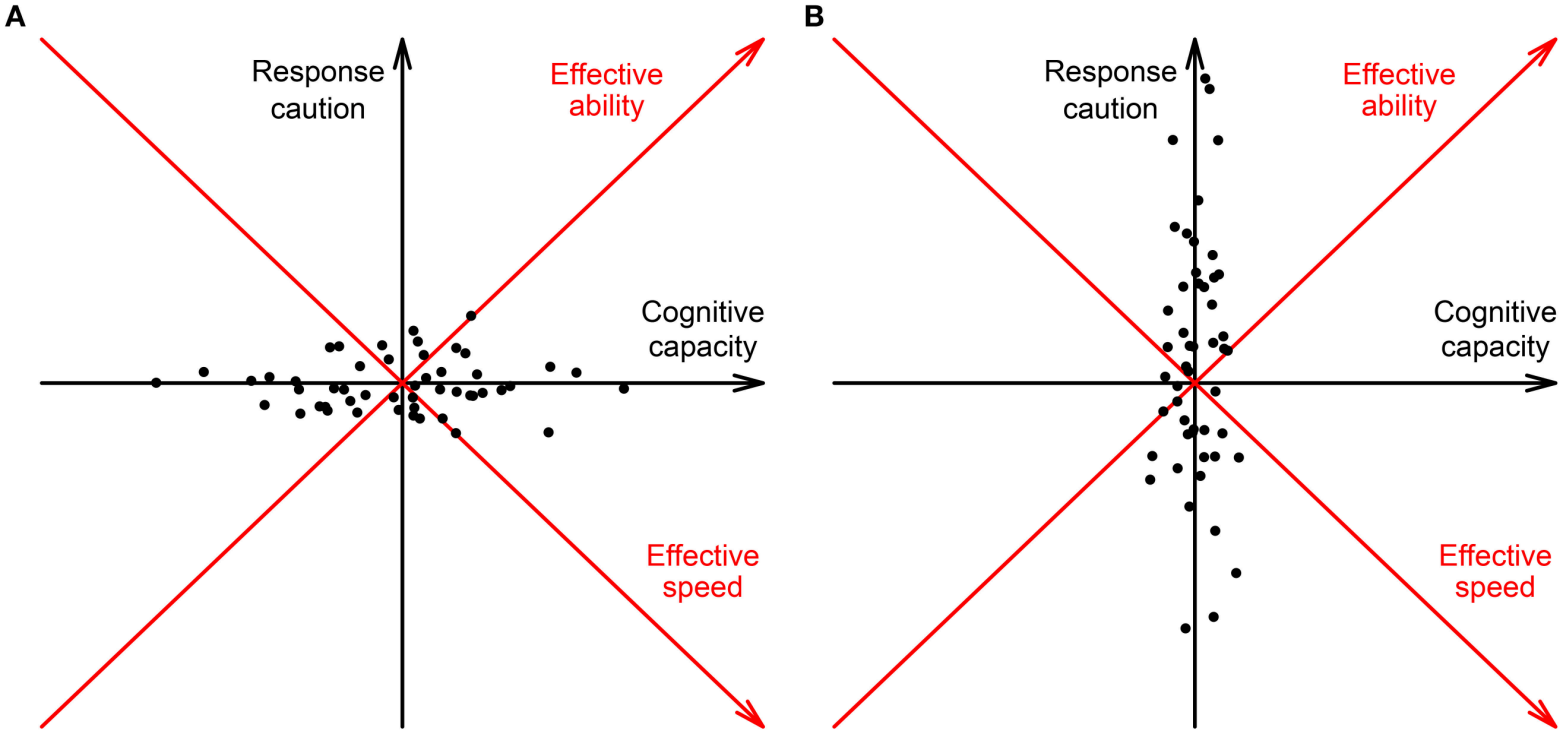
**Hypothetical variation of response caution and cognitive capacity ***within*** a single person during a test: (A)** The variation of cognitive capacity is larger than the variation of response caution, resulting in a positive covariation between effective speed and ability (see the red axes rotated counterclockwise over 45°) and negative conditional dependence between response time and accuracy given the average speed and ability; **(B)** The variation of response caution is larger than the variation of cognitive capacity, resulting in a negative covariation between effective speed and ability (see the red axes rotated counterclockwise over 45°) and positive conditional dependence between response time and accuracy given the average speed and ability. Here, for simplicity we assume that response caution and cognitive capacity vary independently within a person.

To summarize, θ and τ may in practice vary across the test due to variation of response caution, variation of cognitive capacity, or both. Figure 2 shows two examples of this within-person variation. Since cognitive capacity is positively related to both speed and ability, while response caution is positively related to ability and negatively to speed, on the left larger variation of cognitive capacity results in a positive covariation of effective speed and ability (i.e., negative CD) and on the right larger variation of response caution results in a negative covariation of speed and ability (i.e., positive CD). Response caution and cognitive capacity might also co-vary which makes the full picture of CD even more complex.

### 2.2. Variation of difficulty and time intensity across persons

Standard models assume that an item has a constant difficulty and time intensity for all persons, however it is possible that an item's difficulty varies across persons due to idiosyncratic differences. Even persons with the same educational background that are exposed to the same curriculum will have slightly different learning trajectories (e.g., reading book chapters in a different order when preparing for the test). Even larger differences will exist between persons from different educational backgrounds or subpopulations. This will result in some items being relatively more difficult or easy for a particular student compared to its difficulty for the rest of the population. Additionally, if dimensions beyond the ability of interest determine the probability of success (i.e., if there is unmodeled multidimensionality), item difficulty will also be different for persons differing on those additional dimensions. Essentially, such variations in difficulty across persons can be considered to constitute differential item functioning (DIF; see, e.g. Mellenbergh, 1989).

In educational and cognitive tests positive correlation is often found between β and ξ across items (e.g., van der Linden et al., 1999; Klein Entink et al., 2009), so the same pattern can be expected within an item: If a item is more difficult for a particular person than expected (based on β_*i*_), it will also likely require more time to complete (i.e., negative CD).

## 3. Heterogeneous response processes

In the previous subsection we considered situations where the same RP takes place for each person. For some items, however, a response can be obtained in different ways (i.e., using heterogeneous RPs): The RT of person 1 might differ from the RT of person 2 not because they do the *same* thing at a *different* speed, but because they do *different* things (possibly at the *same* speed). For example, the item “1400 − 797 = ?” may be solved either using mental heuristic calculation or using column subtraction on paper. If the different RPs differ both in time intensity and probability of success, CD, either positive or negative, will arise. Whether such differences in the RPs are substantively interesting or should be considered irrelevant noise will depend on the specific differences that are present and the purpose of the test.

One substantively interesting way in which RPs can differ is in the type of processing that a person uses. Here, it has been suggested to distinguish fast processes taken to be relatively automatic and heuristically driven and slow processes which are more controlled and algorithmic (Partchev and De Boeck, 2012; Goldhammer et al., 2014; DiTrapani et al., 2016; Molenaar et al., 2016a). These differences impact both expected RA and RT, resulting in CD that can be positive or negative.

While differences between fundamentally different fast and slow processes are an interesting subject to study for cognitive psychology, other differences in RPs might be solely due to test-taking conditions or particular item features, rather than due to substantively interesting phenomena. This is for example observed when fast random guessing takes place (Schnipke and Scrams, 1997; Wang and Xu, 2015). For most items RA would be higher for normal response behavior than for random guessing (i.e., positive CD). However, if a multiple-choice item contains strong distractors one might observe negative CD, since in that case for low-ability students random guessing might actually be more successful than normal responding.

An alternate form of guessing that may take place is ability-based guessing (San Martín et al., 2006), which is both slower than random guessing and has a higher probability of success. On knowledge-based tests where persons are expected to recognize the correct answer, ability-based guessing results in responses that are both less likely to be correct and have taken more time than responses provided by persons who recognize the correct response option (i.e., negative CD). This direction may reverse for items that contain strong distractors that may trigger incorrect fast responses, when recognition breaks down for many respondents.

When both fast and slow guessing are expected to take place, it is difficult to formulate expectations regarding the direction and strength of CD. This also means that having observed CD on a test where forms of guessing are plausible should not too readily be taken as evidence that a more substantively interesting differentiation between processes is needed to explain the observed CD.

## 4. Toward explanatory models

In this paper we have considered different phenomena which may all lead to CD. Our overview shows that there is much more to the relationship between time and accuracy than can be studied using traditional models assuming CI. Moreover, CD need not be seen as a nuisance to elegant statistical models, but can be seen as a window through which more can be learned about the cognitive processes behind the responses and about individual differences, which makes it highly relevant for psychological research as well as educational testing.

To learn from observed CD one will need to disentangle the possible sources of CD. This may not always be achievable if only RT and RA are available, as different phenomena may result in similar patterns of dependence. However, additional explanatory variables (person, item, or response covariates) might help in determining which particular phenomena are involved. The HM can be extended to include the effects of these covariates on both RT and RA, as illustrated in Figure 1E. Having effects of the covariate on RA and RT that are in the same direction results in positive CD, while effects of opposite directions result in negative CD. To test whether CD observed under the HM can be explained by a particular covariate, one can compare the relative fit of the traditional HM and the model with the effect of the covariate included (Figures 1A,E). Furthermore, one can evaluate whether the match between the observed and the expected correlations between RA and RT improves for the extended model and whether after conditioning on the added covariates CD is present in the extended model (e.g., using posterior predictive checks similar to Bolsinova and Tijmstra, 2016).

For the purpose of extending the HM to explain CD, various relevant covariates can be considered, which should be picked to test hypotheses about the suspected source(s) of CD. Person covariates (e.g., school type or an external measure of reading ability) can be included in the model to test whether CD is due to DIF across subgroups and whether unmodelled multidimensionality leads to negative CD. Item covariates (e.g., whether verbal comprehension is required on a mathematics item) can also be used to test whether CD is due to an unmodeled ability dimension. Similarly, including item position (e.g., 1 for items at the end of the test and 0 otherwise) as a covariate can be used for considering whether differential decrease of response caution added to the model as an additional latent variable is a source of positive CD. Response covariates, such as pupil dilation as a measure of concentration, can be used to test whether negative CD is due to within-person variation of cognitive capacity. Moreover, patterns of eye movements and brain activity, or data from respondents' verbal protocols (i.e., respondents are asked in one way or another about how they solved an item) can be included as response covariates for determining whether CD is due to heterogeneity in the RPs.

Regardless of which specific covariates are considered, a researcher using such an extended HM will have to decide whether to approach these covariates in an exploratory way, or to formulate explicit hypotheses about the direction of their effect that can be tested. While providing the technical details of these different possible modeling approaches goes beyond the scope of this Perspectives paper, we hope that these suggestions provide the reader with an indication of the modeling steps that can be taken to move toward explanatory models for CD.

With the variety of plausible phenomena that may give rise to CD, carefully considering its presence and attempting to understand and model it is important both for the validity of the statistical inferences made based on the responses and for the study of the processes that gave rise to those responses. As our overview of the range of phenomena potentially leading to CD indicates, determining and understanding the exact source(s) of CD likely requires more information than is available from just RA and RT. We argue that considering relevant person, item, and response covariates is a way of moving from descriptive models in which CD is accounted for in a statistical way to explanatory models aimed at obtaining a better understanding of the response phenomena.

## Author contributions

MB and JT wrote the paper, DM and PD provided feedback on the manuscript.

## Funding

The research by DM was made possible by a grant from the Netherlands Organization for Scientific Research (NWO VENI- 451-15-008).

### Conflict of interest statement

The authors declare that the research was conducted in the absence of any commercial or financial relationships that could be construed as a potential conflict of interest.

## Footnotes

^1^A similar mathematical property follows from Ratcliff's unbiased diffusion model (1978), in which given the drift rate and boundary separation the choice made and the time to make it are independent.
